# Nasopharyngeal carriage of individual *Streptococcus pneumoniae* serotypes during pediatric radiologically confirmed community acquired pneumonia following PCV7 introduction in Switzerland

**DOI:** 10.1186/1471-2334-13-357

**Published:** 2013-07-31

**Authors:** Hélène Chappuy, Kristina Keitel, Mario Gehri, René Tabin, Lynda Robitaille, Frederic Raymond, Jacques Corbeil, Veronica Maspoli, Naim Bouazza, Gabriel Alcoba, Laurence Lacroix, Sergio Manzano, Annick Galetto-Lacour, Alain Gervaix

**Affiliations:** 1 Child and Adolescent Department, University Hospital of Geneva, Geneva, Switzerland; 2 Hôpital de l’Enfance, CHUV, Lausanne, Switzerland; 3 Hôpital du Valais, Centre Hospitalier du Centre Valais, Sion, Switzerland; 4 Department of Molecular Medicine, Infectious Disease Research Center, CHUL Research Center and Laval University, Québec, Canada; 5 Unité de recherche clinique Necker Cochin, APHP, Paris, France; 6 Hôpital Necker Enfants Malades, 149 rue de Sèvres 75743, Paris, Cedex 15, France

**Keywords:** *Streptococcus pneumoniae*, Serotypes, Pneumonia, Children, Vaccination

## Abstract

**Background:**

Community-acquired pneumonia (CAP) is a serious cause of morbidity among children in developed countries. The real impact of 7-valent pneumococcal conjugate vaccine (PCV7) on pneumococcal pneumonia is difficult to assess accurately.

**Methods:**

Children aged ≤16 years with clinical and radiological pneumonia were enrolled in a multicenter prospective study. Children aged ≤16 years admitted for a minor elective surgery was recruited as controls. Nasopharyngeal samples for PCR serotyping of *S*. *pneumoniae* were obtained in both groups. Informations on age, gender, PCV7 vaccination status, day care/school attendance, siblings, tobacco exposure were collected.

**Results:**

In children with CAP (n=236), 54% of the nasopharyngeal swabs were PCR-positive for *S*. *pneumoniae* compared to 32% in controls (n=105) (p=0.003). Serotype 19A was the most common pneumococcal serotype carried in children with CAP (13%) and in controls (15%). Most common serotypes were non-vaccine types (39.4% for CAP and 47.1% for controls) and serotypes included only in PCV13 (32.3% for CAP and 23.5% for controls). There was no significant difference in vaccine serotype distribution between the two groups. In fully vaccinated children with CAP, the proportion of serotypes carried only in PCV13 was higher (51.4%) than in partially vaccinated or non vaccinated children (27.6% and 28.6% respectively, p=0.037).

**Conclusions:**

Two to 4 years following introduction of PCV7, predominant *S. pneumoniae* serotypes carried in children with CAP were non PCV7 serotypes, and the 6 new serotypes included in PCV13 accounted for 51.4% of carried serotypes in fully vaccinated children.

## Background

Community-acquired pneumonia (CAP) is a serious cause of morbidity among children in developed countries and has a considerable impact on health care systems. In children <5 years old, *Streptococcus pneumoniae* is considered the most important bacterial pathogen causing pneumonia with an incidence rate of 462 per 100,000 in Europe [1].

Introduction of the 7-valent pneumococcal conjugate vaccine (PCV7) at the beginning of this century provided significant protection against invasive diseases such as meningitis and septicemia. Vaccination also allowed reduction in nasopharyngeal carriage of vaccine-serotype pneumococci among vaccinated children, but induced an increased carriage of nonvaccine serotypes (serotype replacement) [2-6].

After PCV7 was added to universal immunization programs, a reduction of 13% to 65% in the hospitalization rate for pneumonia was noted in the United States [7], Canada [8], England [9], and Italy [10]. However, the real impact of this vaccine on pneumococcal pneumonia is difficult to assess accurately. Identifying the cause of a lower respiratory tract infection remains a challenge for a number of reasons: adequate samples are difficult to obtain, and differentiation between infection and colonization cannot always be made. Michelow et al. [11] reported that using a sophisticated armentarium of blood PCR, serology and culture for 6 viruses and bacteria, a pathogen could be detected in 79% of children with pneumonia. In a recent study we performed in Switzerland, we showed that *S. pneumoniae* was recovered in 45% of cases of all pneumonia, and in 71% of pneumonia where a bacteria was evidenced [12].

Serotype determination in pneumococcal pneumonia derives mainly from blood or pleural fluid in children with invasive pneumonia. However, most cases of pneumonia are not bacteremic [12], resulting in difficulty to determine serotypes causing non invasive pneumonia. It is commonly accepted that carriage of *S. pneumoniae* precedes invasive disease and infections such as otitis media and pneumonia [13-15]. Although the presence of *S. pneumoniae* in the nasopharynx during pneumonia does not prove its causative role, a pneumococcal disease is likely to be caused by the serotype present in the nasopharynx. A recent Israeli study estimated pneumococcal serotype-specific disease potential in pediatric community-acquired alveolar pneumonia, by comparing nasopharyngeal pneumococcal carriage during disease to carriage in healthy children [16]. They concluded that serotypes 1, 5, 7F, 9V, 14, 19A, and 22F have a higher disease potential for childhood pneumonia than do serotypes 6A, 6B, 23A, and 35B. However, this study was performed before routine implementation of the heptavalent pneumococcal vaccine. Monitoring the changes in pneumococcal nasopharyngeal carriage is important and can provide early, relevant information on the vaccine effect, particularly concerning the identification of serotypes that may substantially contribute to pneumococcal diseases in the post-vaccine era. To our knowledge, there is no data on the effect of PCV7 on nasopharyngeal pneumococcal colonization among children with CAP in Switzerland, where the primary vaccine series is administered during infancy and the booster dose at 12–15 months of age.

The aim of this two year prospective study was to document the rate of nasopharyngeal carriage of pneumococci in Swiss children suffering from CAP, compared to healthy children and to assess serotype distributions in CAP and the potential impact of PCV13.

## Methods

### Setting

A multicenter prospective cohort study was conducted in the Pediatric Emergency Department of 3 major hospitals in Switzerland (Geneva, Lausanne and Sion) from January 2008 to September 2010. PCV7 has been available in the routine vaccination program since January 2006 in Switzerland. Around 70% of infants of 2years of age were fully vaccinated (3 doses) with PCV-7 at the time of the study [17]. PCV13 has not been licensed in Switzerland before 2011.

### Subjects studied

We postulated that populations with high rates of *S. pneumoniae* carriage are particularly valuable for studying the impact of vaccination programs. The study population was defined to maximize the probability of detecting an impact on carriage of pneumococcal serotypes.

Cases were defined as children aged ≥2 months and ≤16 years seen at the pediatric emergency room with fever (>38C) and cough, tachypnea or respiratory distress, and abnormal lung infiltrates on Chest X-ray (CXR). Children with previously diagnosed chronic lung or heart diseases, immunodeficiency syndromes and hospital-acquired pneumonia were excluded. The pediatric radiologist assessed the CXR, unaware of the interpretation of other investigators, nor of the clinical diagnosis. Definitive cases were those confirmed by the radiologist according to the WHO criteria [18]. Blood tests including White blood cell (WBC), C-reactive protein (CRP), Procalcitonin (PCT) were obtained.

To determine the differences in serotype-specific carriage rates in CAP children versus healthy children, a control group was enrolled during the same period. Children aged ≥2 months and ≤16 years, admitted to the pediatric surgery division for a minor elective surgery (inguinal hernias, circumcision, hypospadias) were recruited as controls. Children with any history of chronic disease or lower respiratory tract infection within the past 3 weeks were excluded.

Information on age, gender, PCV7 vaccination status, day care/school attendance, siblings, tobacco exposure was collected in both groups.

Children were considered to be fully vaccinated if they had completed 3 doses of the PCV7 at 2, 4 and 12 months per the routine Swiss childhood vaccination schedule. Children were considered to be incompletely vaccinated if they had started but not completed the 3 doses.

Ethical approval was obtained from the Research Ethics Committees of the University Hospitals of Geneva, University Hospital of Lausanne and Hospitals of Valais. Informed consent was obtained from parents of participating children before recruitment.

### Laboratory methods

Nasopharyngeal samples for *S. pneumoniae* PCR serotyping were obtained systematically in both groups with a flexible dacron-tipped swab (eSwab, Copan Inc., Brescia, Italy), which was introduced into the nostrils and advanced until resistance was found. They were taken at the time of admission and were frozen at −80C in Amies medium in order to be subsequently analyzed. Detection and identification of *S. pneumoniae*, was done using a *Streptococcus pneumoniae* serotyping assay that genotypes eight capsular genes in order to identify pneumococcal serotypes. The assay was performed on the INFINITI analyzer (Autogenomics, Carlsbad, CA) and consists in five main steps: multiplex PCR for target genes amplification, primer extension and labeling for specific polymorphisms identification, microarray hybridization, fluorescence reading and interpretation by expert system. The combination of nucleotides typed is compared to a database of sequences in order to identify the serotype of the sample. The assay also detects *autolysin* (*lytA*), *pneumolysin* (*ply*) and an internal control, which use the M13 bacteriophage. In silico, this assay allowed the precise identification of 52*S. pneumoniae* serotypes using a single assay, including the serotypes covered by PCV13. Fifty subtypes were sequenced verified [19]. The limit of detection for serotyping was one hundred genome equivalents and was consistent for all serotypes.

In the present study, the serotypes included in the 7-valent pneumococcal conjugate vaccine (4, 6B, 9V, 14, 18C, 19F, and 23F) are defined as PCV7 and the serotypes included in addition to PCV7 in the 13-valent vaccine (1, 3, 5, 6A, 7F and 19A) are defined as PCV13. The other serotypes are defined as non vaccine type, or not determined.

### Statistical analysis

Population description was made through usual tools, using proportions for categorical data; mean and 95% confidence interval (CI) on the mean. Normality assumption was checked by quantile-quantile plots and Shapiro-Wilks’ test. Host and environmental factors were compared between children with and without CAP using t-tests for continuous variables (age) and Pearson Chi-square analyses for categorical variables (day-care attendance, siblings, gender, PCV7 vaccination status). Means and 95% confidence interval (CI) for age, CRP, PCT, and WBC were expressed. Nasopharyngeal carriage of *S. pneumoniae* was compared between children with and without CAP using Pearson’s Chi-square analyses and Student’s T test for quantitative data. *S. pneumoniae* serotypes carried in CAP patients and healthy controls was studied through multiple regression analysis, adjusting for age, gender and PCV7 vaccination status to ensure the comparability between the two groups. PCV7 vaccination status in CAP patients and healthy controls as well as the influence of PCV7 vaccination status on *S. pneumoniae* carriage has been studied using a multivariate analysis including age and gender as adjusting variables. A 2-tailed cut-off of p<0.05 was considered a statistically significant difference.

Data were analysed using the statistical analysis package SPSS for Windows, version 15.0.

## Results

### Study population

During the study period, 236 patients with CAP and 105 control patients were enrolled.

General characteristics of the study cohort are described in Table 1. The mean age of the CAP group was 4.7 yrs (95% CI: 0.7-14) compared to 6.0yrs (95% CI: 1.2-15.1) for the control group (p<0.001). The proportion of male children was higher in the control group compared to the CAP group (p=0.004). This is explained by the high frequency of male-specific surgical procedures in the control group (orchidopexia, inguinal hernias, circumcision, hypospadias). Among children with CAP, 26% had received three doses of PCV7 compared with 13% in the control patients (p=0.03). Incomplete vaccination was found in 17 patients with CAP (7.9%) versus 3 control patients (3.1%). One hundred forty two patients with CAP (65.7%) were non vaccinated versus 81 control patients (83.5%). The difference was not significant after adjustment to age and gender (p=0.08; data not shown).

**Table 1:** Characteristics of the two groups of participants

**Variables**	**CAP (n=236)**	**Healthy (n=105)**	**p-value**	Adjusted p-values*
	Mean (95% CI)			
**Age in years**	4.7 (0.7-14)	6 (1.2-15.1)	<0.001	-
Range (min-max)	0.24-15.3	0.20-16.3		
**CRP level mg/L**	102 (9.6-315.2)	NA	-	-
WBC count, cells *10^**9**^**/L**	15.4 (4.5-35.6)	NA	-	-
**PCT**	7.8 (0.06-57.8)	NA	-	-
	n (%)			
**Male**	116/229 (51)	71/105 (68)	0.004	-
**Fully vaccinated with PCV-7**	57/216 (26)	13/97 (13)	0.004	-
**Day care/school attendance**	167/226 (74)	82/102 (80)	0.20	0.94
**Number of siblings >1**	152/223 (68)	76/103 (74)	0.30	0.56
**Positive tobacco exposure**	64/219 (29)	39/100 (39)	0.08	0.13
**Nasopharyngeal SP_ PCR positive**	127 (54)	34 (32)	<0.0001	0.003

*CAP* indicates Community-acquired pneumonia; *SP*, *Streptococcus pneumoniae*; *n*, number; *CRP*, C reactive protein; *WBC*, white blood cell; *PCT*, Procalcitonin; *NA*, not applicable.

* Chi-Square test adjusted by age, gender and PCV7 vaccination status (fully, partially or not).

The presence of siblings, cigarette smoke exposure, and day care/school attendance were similarly distributed between children with and without a CAP.

### Pneumococcal serotypes in the nasopharynx

**Figure 1:**
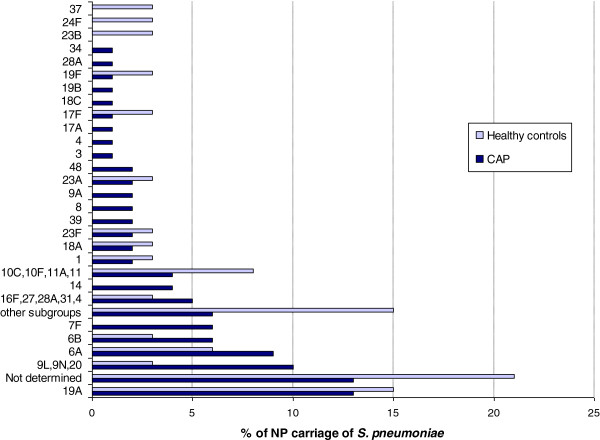
**Nasopharyngeal carriage of *S. pneumoniae *individual serotypes in children, CAP versus healthy controls.**

In children with CAP, 54% of the nasopharyngeal swabs were PCR-positive for *S. pneumoniae* compared to 32% in healthy children (p=0.003; Table 1). Concerning antibiotics use before ED visit, we have data for 122/127 patients with CAP. Only 20 patients received penicillin before their ED visit. There was no significant difference on serotypes (p=0.46). The most common pneumococcal serotypes carried in children with CAP were 19A (13%), 6A (9%), 6B (6%) and 7F (6%). For control patients, serotype 19A (15%) was the most common (Figure 1).

In both groups, PCV7 serotypes were infrequently found (n=22, 13.7%). Most common serotypes were non-vaccine types (39.4% for CAP and 47.1% for control patients) and serotypes included only in PCV13 (32.3% for CAP and 23.5% for control patients). *S. pneumoniae* serotypes were not determined with the assay we used in 20.6% (n=7) of control patients and in 13.4%, (n=17) in children with CAP. There was no significant difference in vaccine serotype distribution between the two groups (Table 2).

**Table 2:** ***S. pneumoniae *serotypes carried in CAP patients and healthy controls**

	**CAP**	**Healthy controls**	**p-value**	Adjusted p-value*	
	**(n=127)**	**(n=34)**		**(1)**	**(2)**
**SEROTYPES**			0.43	0.47	0.6
**Included in PCV 7**	19 (14.9%)	3 (8.8%)			
**Included only in PCV 13 (6 serotypes)**	41 (32.3%)	8 (23.5%)			
**Non vaccine type**	50 (39.4%)	16 (47.1%)			
**Not determined**	17 (13.4%)	7 (20.6%)			

* Chi-Square test adjusted by age, gender (1) and by age, gender and PCV7 vaccination status (2).

**Figure 2:**
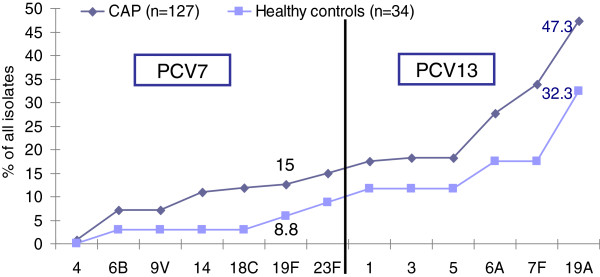
Cumulative potential serotype coverage of PCV7, PCV13 of serotypes in CAP patients and healthy controls.

Figure 2 show that PCV13 would extend the potential coverage to 47.3% of CAP and 32.3% of control patients. For CAP cases, the addition of serotypes 19A, 6A and 7F included in PCV13 is important, because it would add 13.4%, 9.4% and 6.3% to the overall serotype coverage beyond PCV7.

### Effect of the PCV7 vaccination status on CAP

As shown in Table 3, serotype distribution of *S. pneumoniae* carried in CAP patients was significantly influenced by PCV7 vaccination status (p=0.037). In fully vaccinated children with CAP, the proportion of serotypes carried only in PCV13 was higher (51.4%) than in the others groups: 27.6% in patients who had received one or two doses and 28.6% in non-vaccinated children. The other half of patients carried nonvaccine types. Surprisingly, two fully vaccinated three years old children with CAP carried PCV7 serotypes: 14 and 23F. For non-vaccinated children with CAP, 57.1% of them carried a non vaccine type and 14.3% a PCV7 serotype (Table 3).

**Table 3:** **Serotype distribution of *S. pneumoniae *carried in CAP patients by PCV7 vaccination status**

	**PCV7 vaccine status**			**Total**
**SEROTYPES**	None	1 or 2 injections	Fully	
**Included in PCV 7**	9 (14.3%)	8 (27.6%)	2 (5.7%)	19 (15%)
**Included only in PCV 13 (6 serotypes)**	18 (28.6%)	8 (27.6%)	18 (51.4%)	44 (34.6%)
**Non vaccine type**	36 (57.1%)	13 (44.8%)	15 (42.9%)	64 (50.4%)
**Total**	63 (100%)	29 (100%)	35 (100%)	127 (100%)

Chi-Square=10.2 (p=0.037).

PCV7 vaccination status did not show any effect on nasopharyngeal pneumococcal carriage between the two groups (data shown in Table 4).

**Table 4:** **Influence of PCV7 vaccination status on *S. pneumoniae *carriage**

	**Nasopharyngeal SP_PCR +**	**Nasopharyngeal SP_PCR -**	**p-value**	Adjusted p-value*
	**(n=151)**	**(n=162)**		
**PCV7 Vaccination status**			0.046	0.98
Fully vaccinated	40 (26.5%)	30 (18.5%)		
Partially vaccinated	13 (8.6%)	7 (4.3%)		
None	98 (64.9%	125 (77.2%)		

*Chi-Square test adjusted by age, gender.

## Discussion

Community-acquired pneumonia is one of the most significant contributors to the morbidity and mortality of pneumococcal disease. Detailed information on the etiology of CAP is required for the initiation of an appropriate treatment and for the evaluation of preventive measures such as pneumococcal conjugate vaccines. We have found that 2 to 4 years following introduction of PCV7 in Switzerland, predominant *S. pneumoniae* serotypes carried in children with CAP were non PCV7 serotypes, and that the 6 new serotypes included in PCV13 accounted for 51.4% of carried serotypes in fully vaccinated children.

In our study, positive nasopharyngeal pneumococcal PCR was found in half the children with CAP versus in only one third of control patients. For control patients, these results were similar to a recent American study where pneumococcal carriage persisted in 30% of young children in the post-PCV7 era [20]. As a rule, *S. pneumoniae* as the causative serotype is found in the nasopharynx during non invasive diseases like otitis media or pneumonia [13-15,21].

We found that pneumococcal serotypes 19A and 6A were the most commonly carried serotypes in our study population. These data are consistent with recent studies from the US [20,22] and from Australia [23]. Other studies showed that serotypes 6A, 6B and 19A were frequently found during pneumonia [16,24]. The PROTEKT US study [25] showed that the non vaccine serotypes 19A (19.0% of all isolates) and 6A (7.8%) were among the most frequently found serotypes in respiratory tract isolates from children with non invasive diseases, 4 years after vaccine introduction. Serotypes 19A together with 7F are among the major causes of pneumococcal empyema in the post-PCV7 era [26,27]. Serotype 19A is currently the major cause of invasive pneumococcal disease [6,28-30], mastoiditis, and refractory acute otitis media (AOM) in infants and children. Moreover, it may be multidrug resistant [26,31,32].

We observed that following introduction of PCV7, carriage of PCV7 serotypes was limited (10-15%) in both children with and without CAP. Nevertheless, this is higher than previously described in US studies showing a reduction in carriage of PCV7 serotypes from 36% in 2001, to 3% 6 to 8 years after introduction of PCV7 [20,22] and in an Australian study 2 to 4 years after introduction of PCV7 showing a result of 3% [23]. It may be explained by the low vaccination coverage in our study population compared to these countries [17]. In our study, among the children with CAP, 26% had received three doses of PCV7 compared with 13% of the control patients.

In fully vaccinated children with CAP, the proportion of non-PCV7 serotypes carried was predominant. In this group, half of the serotypes were those included in the new PCV13 vaccine and almost one half were non PCV13 serotypes. The importance of non vaccine type (neither PCV7 nor PCV13) existed likewise in non vaccinated children with CAP. An important finding of almost all trials concerning the conjugate pneumococcal vaccine is not only a significant reduction in nasopharyngeal carriage of pneumococci serotypes included in PCV7 but also a concomitant increase in carriage of non-PCV7 serotypes, in 39% up to 79% of cases [33,34]. In recent years, data showed the same phenomenon for invasive diseases [6,35]. Marchese et al. [36] using blood PCR, showed that more than 47% of Bacteremic pneumonia cases in children less than 5 years old were due to non-PCV7 serotypes. Concerning non invasive diseases, Casey et al. [22] reported that non-PCV7 serotypes like 19A and 6A were among the most common causes of pneumococcal otitis media in the post-PCV7 era. However, care should be taken when assuming a causal relationship between PCV7 vaccination and an increase in 19A diseases, since recent reports describe an increase in 19A even in countries without routine PCV7 vaccination [37,38]. A French study [39], showed that 19A carriage was the first leading non-vaccine serotype. It did not increase significantly (between 8% and 10%) over a 5-year period (2001-2006) and was comparable in both vaccinated and non-vaccinated populations with acute otitis media.

In February 2010, a novel PCV13 was licensed for US children aged 6weeks to 71months. PCV13 includes serotypes 1, 3, 5, 6A, 7F, and 19A in addition to the serotypes contained in PCV7. This should limit the spread of serotype 19A by providing direct protection. Our study showed that the 6 new serotypes of PCV13 would extend the coverage in 47% of CAP and in 32% of control patients. In our population, the addition of serotypes 19A, 6A and 7F included in PCV13 would add 13.4%, 9.4% and 6.3% to the overall serotype coverage beyond PCV7. Shouval et al. [40] showed that PCV13 extended the coverage to 79% of acute otitis media and 67% of carriage groups versus 54% and 46% respectively, with PCV7. The scenario for prevention of acute otitis media and non bacteremic pneumonia is less clear than in invasive diseases. Indeed, the differences in “invasive capacity” among serotypes with regard to their ability to ascend the Eustachian tube or overcome host defenses of the respiratory tract appear to be small. However, increased serotype coverage of PCV13 is expected to have a substantial public health impact on infectious diseases. Chuck AW et al. [41] developed a simulation model for an entire population, providing vaccine to children less than 2 years of age. With PCV 13-valent vaccine introduction and a herd effect, pneumonia cases would reduce from 373 per 100,000 persons with PCV-7 coverage only to 336.2 cases. Herd protection can have an important role in the reduction of disease in both vaccinated and unvaccinated people, through decrease in the nasopharyngeal carriage of vaccine serotypes [42,43].

This study has several limitations. It revealed considerable diversity in serotypes within pneumococci retrieved from cases of CAP and control patients. As a consequence, many serotypes were not present in sufficient numbers to furnish statistical power to detect an association with carriage or CAP. We cannot draw conclusions on how the not determined nasopharyngeal serotypes might have impacted the serotype distribution in our study. There are differences in the distribution of risk factors between the two groups of children, which may influence the comparison between these groups. However, these factors were taken into account in our statistical analyses.

## Conclusion

In conclusion, although PCV7 has the potential to substantially decrease pneumococcal diseases, carriage and spread, our results strongly suggest that PCV13 could have a significant added benefit in reducing pneumococcal pneumonia burden in Switzerland. Bacteriologic surveys of community-acquired pneumonia remain necessary.

## Abbreviations

CAP: Community-acquired pneumonia; CRP: C-reactive protein; CXR: Chest X-ray; IPD: Invasive Pneumococcal Disease; NP: Nasopharyngeal; PCT: Procalcitonin; PCV7: Heptavalent pneumococcal conjugate vaccine; PCV13: Thirteen valent pneumococcal conjugate vaccine; WBC: White blood cell.

## Competing interests

The authors declare having no conflict of interest that are directly relevant to the content of this study. This study was sponsored in part by Wyeth /Pfizer Pharmaceuticals Inc. as a part of an ongoing larger study on Prevenar (PCV7) vaccine.

## Authors’ contribution

A G. designed this study and obtained research funding. L R., F R., J C. were in charge of the serotyping method. H C. and N B. were in charge of all the data analyses. H C. took the lead in reviewing the literature, crosschecking references and drafted major portions of the initial manuscript. A G., M G., G A., V M., K K., L L., S M., A G-L. recruited patients and helped in writing the final manuscript. All authors read and approved the final manuscript.

## Pre-publication history

The pre-publication history for this paper can be accessed here:

http://www.biomedcentral.com/1471-2334/13/357/prepub
